# Viral metagenomics reveals diverse viruses in the fecal samples of children with acute respiratory infection

**DOI:** 10.3389/fmicb.2025.1564755

**Published:** 2025-04-07

**Authors:** Pan Xu, Chunduo Pan, Minli Yuan, Ying Zhu, Shanjie Wei, Hongyan Lu, Wen Zhang

**Affiliations:** ^1^Department of Pediatrics, The Affiliated Hospital of Jiangsu University, Jiangsu University, Zhenjiang, Jiangsu, China; ^2^Department of Microbiology, School of Medicine, Jiangsu University, Zhenjiang, Jiangsu, China

**Keywords:** acute respiratory infection, children, viral metagenomics, fecal samples, virus evolution

## Abstract

**Introduction:**

Changes in the gut microbiome have been associated with the development of acute respiratory infection (ARI). However, due to methodological limitations, our knowledge of the gut virome in patients with ARIs remains limited.

**Methods:**

In this study, fecal samples from children with ARI were investigated using viral metagenomics.

**Results:**

The fecal virome was analyzed, and several suspected disease-causing viruses were identified. The five viral families with the highest abundance of sequence reads were *Podoviridae*, *Virgaviridae*, *Siphoviridae*, *Microviridae*, and *Myoviridae*. Additionally, human adenovirus, human bocavirus, human astrovirus, norovirus, and human rhinovirus were detected. The genome sequences of these viruses were respectively described, and phylogenetic trees were constructed using the gene sequences of the viruses.

**Discussion:**

We characterized the composition of gut virome in children with acute respiratory infections. However, further research is required to elucidate the relationship between acute respiratory infection and gut viruses.

## 1 Introduction

Acute respiratory infections (ARIs) exhibit high prevalence in pediatric populations and pose a significant threat to global child health. According to statistics, globally, 502,000 children die from respiratory infections annually, among which 254,000 deaths occur in countries with a low Socio-Demographic Index (Bender et al., 2024). The majority of ARIs are caused by viruses, including influenza virus (IFV), respiratory syncytial virus (RSV), human rhinovirus (HRV), human parainfluenza virus (HPIV), human adenovirus (HAdV), human coronavirus (HCoV), human bocavirus (HBoV), and human metapneumovirus (HMPV) (Jain et al., 2015; Li Z. et al., 2021). Accurate pathogen detection remains critical for clinical diagnosis, therapeutic management, and epidemiological control. Traditional virus detection methods, including virus isolation and identification, PCR, and ELISA, are complicated, time-consuming, and unable to cover all viruses.

The development of viral metagenomics technology provides an unbiased, high-throughput solution for virus analysis. Compared with traditional methods, this technology can simultaneously identify all viral sequences in a sample, including unknown or rare pathogens, thus revealing the complex structure of the gut virome (Capobianchi et al., 2013). In addition, viral metagenomics technology plays an important role in epidemiological work. By integrating clinical data with information of virome, this technology can analyze the epidemic patterns, transmission chains, and risk factors of viruses, providing key scientific evidence for optimizing epidemic monitoring or prevention and control strategies, as verified in the prevention and control of various viral diseases such as influenza, SARS-CoV-2, and Ebola virus (Gauthier et al., 2023). In children with acute respiratory infections, Ogunbayo et al. (2023) detected viral pathogens in 82 out of 84 patients using metagenomics technology and identified the pathogens in 9 previously undetected or missed cases, which was significantly higher than the results of traditional detection.

Emerging evidence implicated the connection between gut microbiota and respiratory health, which play a crucial role in maintaining normal immune function and resisting infections. Lu et al. (2021) demonstrated that the gut virome of patients with severe acute respiratory syndrome coronavirus 2 (SARS-CoV-2) infection in 2019 was significantly altered. Leal Rodríguez et al. (2024) observed an association between specific gut viruses in infants and the subsequent development of preschool asthma. Moreover, an analysis of COPD patients revealed changes in the gut virome, which were found to be correlated with alterations in pulmonary ventilation function and viral function (Wang et al., 2024).

In reality, research on the gut virome of children with ARI has not been reported yet. The aim of this study is to use viral metagenomics technology to analyze gut virome in the fecal samples of children with ARI, and combine with known epidemiological data to reveal the possible prevalence of some viruses, advancing our understanding of the gut viral community in children with acute respiratory infections.

## 2 Materials and methods

### 2.1 Sample collection

To exclude the influence of SARS-CoV-2 and mycoplasma pneumoniae, during April to September 2023, we collected fresh fecal samples from 121 children less than 12 years old. These children presented with at least two of the following acute respiratory symptoms: fever, cough, sore throat, nasal congestion, and rhinorrhea. These symptoms were documented at the Affiliated Hospital of Jiangsu University. To prevent potential cross-contamination, all samples were collected with sterile disposable materials and stored in 1.5-mL sterile enzyme-free centrifuge tubes. After collection, these samples were transported to the laboratory on dry ice via a cold chain, and stored in a refrigerator at −80°C until further analysis. The patients included in this study had signed informed consent forms, which had been approved by the Medical Ethics Committee of the Affiliated Hospital of Jiangsu University under the number KY2023K0805.

### 2.2 Sample preparation and library construction

In this study, 121 fresh fecal samples were randomly assigned to 22 libraries, encompassing 10 single-sample libraries (Humanfe170 - Humanfe179) and 12 mixed-sample libraries (Humanfe180 - Humanfe191). The detailed group information is available in Supplementary Table 1. Before constructing libraries, the samples were required to undergo a series of preprocessing steps. Once the samples were thawed, 500 μL of DPBS buffer was added, and the mixture was shaken repeatedly three times and subsequently centrifuged (12,000 *g*, 4°C) for 5 min to obtain the supernatant. The samples were then mixed in accordance with the group information (Supplementary Table 1), with a total volume of 500 μL. The eukaryotic- and bacterialcell-sized particles were eliminated by filtration through a 0.45 μm centrifugal filter (Millipore) at 4°C and 12,000 *g* for 5 min. The viral-enriched filtrate was collected and treated with nuclease enzymes (Qiagen) at 37 C for 60 min to digest unprotected nucleic acids (Zhang et al., 2014a; Zhang et al., 2014b). A QIAamp Viral RNA Mini Kit (QIAGEN) was used to extract nucleic acid according to the manufacturer’s instructions, and reverse transcription was performed with six random base primers and SuperScript III reverse transcriptase (Invitrogen). The second strand of cDNA was then synthesized using the Klenow fragment DNA polymerase. The Nextera XT DNA Sample Preparation Kit (Illumina) was utilized to construct the sequencing library, and the Miseq second-generation sequencing platform was used for sequencing. Each library was sequenced with 250 bp paired-end reads and dual barcodes (Xiao et al., 2020).

### 2.3 Bioinformatics analysis

The Illumina Vendor software (bcl2fastq2 v2.20) was employed to interpret the 250 bp paired-end reads generated by the Miseq platform. Subsequently, the data processing was conducted using an in-house analysis pipeline operating on a 32-node Linux cluster. We used the default settings of the Bowtie2 v2.5.2 (Langmead and Salzberg, 2012) software and screened the prokaryotic and eukaryotic gene sequences from the raw sequencing results using the complete genomes of bacteria and fungi in GenBank version 263 from the National Center for Biotechnology Information (NCBI) as reference sequences. The Phred software was adopted to trim the low-quality tails and repetitive sequences at both ends of the sequences, with a quality score of 10 as the threshold. VecScreen software was applied with default parameters to remove the adapter and primer sequences. Then, the trimmed data were assembled by means of the ENSEMBLE software to obtain the maximum contigs and singletons. The assembled contigs and singletons were subsequently compared with the internal virus protein database via BLASTx, with an *E*-value cutoff of less than 10−5. The internal viral protein database was compiled using the NCBI virus reference proteome, to which viral protein sequences from the NCBI nr fasta file were added (based on the annotation taxonomy in the Virus Kingdom). (Altschul et al., 1997; Deng et al., 2015).

### 2.4 Statistical analysis

The statistical analysis in this experiment was carried out using MEGAN v6.25.6 (Huson et al., 2016) and R v4.3.3. The components of the 22 libraries were standardized and analyzed with the aid of MEGAN. The composition of the virus community was visualized by employing the pheatmap and ggplot2 software packages within R v4.3.3.

### 2.5 Phylogenetic analysis

To obtain the complete genomes or longer contigs, each viral contig was used as a reference for mapping the raw data using the Low Sensitivity/Fastest parameter in Geneious v2024.0.5. Assembled genome or contigs were compared to reference strains in the NCBI Nucleotide database (Release 263) using BLASTn with a sequence identity threshold of >95% and an *e*-value cutoff of <1e−5. Reference strains were prioritized based on clinical relevance and genomic completeness. Viral nucleic acid sequences obtained through bioinformatics analysis, along with the best-matched sequences from the NCBI GenBank database and representative sequences of the corresponding virus family, were used for a systematic phylogenetic analysis. The relevant sequences were aligned using the default settings of MUSCLE (Edgar, 2004) in MEGA v11.0.13 (Kumar et al., 2018). After trimming, the phylogenetic trees were constructed using MrBayes v3.2.7 (Ronquist et al., 2012). In MrBayes, two Markov Chain Monte Carlo (MCMC) sampling runs were conducted simultaneously, and the analysis was terminated when the average standard deviation of split frequencies was less than 0.01. The first 25% of the trees were discarded as burn-in. The phylogenetic trees were visualized using FigTree v1.4.4 and further refined with Adobe Illustrator 2024 v28.5.0.

## 3 Results

### 3.1 Overview of viral metagenomics

**FIGURE 1:**
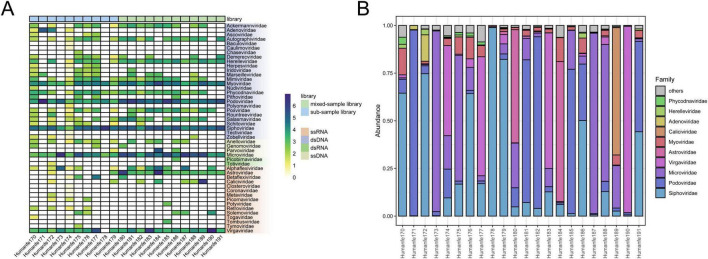
Taxonomic analysis of fecal virome detected in ARI children on the family level. **(A)** Heatmap was constructed after transforming read counts per virus family in individual libraries on a log10 scale. Nucleic acid types and virus families are annotated with different colors (see color legend). **(B)** Bar chart by virus database showing the relative proportion and taxonomy based on viral families.

On the Illumina MiSeq platform, these 22 libraries altogether generated 67,118,096 raw sequencing reads. Of these, 45,991,584 reads (68.52%) were identified as viral sequences through BLASTx searches against the non-redundant protein database of GenBank. Detailed information regarding the libraries was provided in Supplementary Table 1. The identified viral sequences were categorized into 47 families, comprising 26 dsDNA virus families, 4 ssDNA virus families, 2 dsRNA virus families, and 15 ssRNA virus families. The heatmap (Figure 1A) showed that the most abundant viral families in the dataset were *Podoviridae* (31.1%), *Virgaviridae* (21.5%), *Siphoviridae* (10.7%), *Microviridae* (15.8%), and *Myoviridae* (2.5%). Additionally, we ranked the top ten viral families with the highest abundance in each library in descending order: *Siphoviridae*, *Podoviridae*, *Microviridae*, *Virgaviridae*, *Astroviridae*, *Myoviridae*, *Caliciviridae*, *Adenoviridae*, *Herelleviridae*, and *Phycodnaviridae* (Figure 1B). We noticed variations in the proportions of viral families across different libraries. The relative abundance plot (Figure 1B) showed that the composition of gut viruses was roughly the same among the 10 single-sample libraries (Humanfe170 - Humanfe179), but there were significant differences in the proportions of the same viruses in different libraries. Furthermore, we identified disease-associated viruses in the majority of the libraries, including *Adenoviridae*, *Herpesviridae*, *Anelloviridae*, *Parvoviridae*, *Astroviridae*, *Caliciviridae*, and *Picornaviridae*.

### 3.2 Human adenovirus belonging to *Adenoviridae*

**FIGURE 2:**
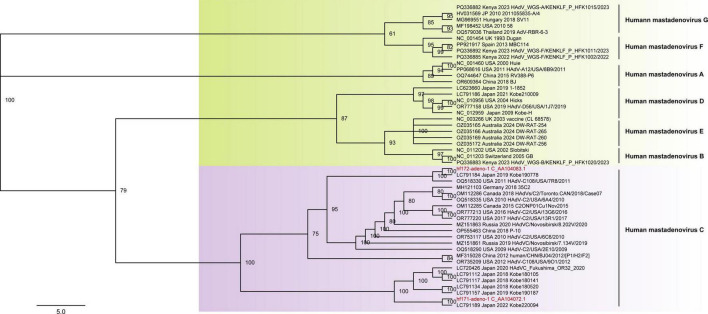
The phylogenetic analysis of HAdVs identified in this study. The phylogenetic trees were constructed based on the nearly complete genome of HAdVs that included the reference strains of seven different genotypes. Viruses identified in this study were marked in red font.

In this study, human adenoviruses (HAdVs) were identified from four libraries (Humanfe170-172, 174). Two nearly complete HAdV genomes were assembled using Geneious 2024.0.5 and named hf171-adeno-1 and hf172-adeno-1, respectively (Supplementary Table 2). The genome lengths of these HAdVs are 36,132 nt and 36,069 nt, encoding 30 proteins. The GC content is 55.2% for both genomes. To investigate the relationship between these HAdVs and other known adenoviruses, BLASTn search in NCBI revealed that hf171-adeno-1 shared the highest identity of 99.54% with the strain Kobe180141 (LC791117), isolated from Japan in 2018, and hf172-adeno-1 exhibited maximum identity of 99.79% with the strain Kobe190778 (LC791184), isolated from Japan in 2019. Phylogenetic trees were constructed based on the full genomes of seven different human adenovirus species, which served as reference strains. The results indicated that hf171-adeno-1 and hf172-adeno-1 clustered with human adenovirus C to form a distinct branch (Figure 2).

### 3.3 Human bocavirus belonging to *Parvoviridae*

**FIGURE 3:**
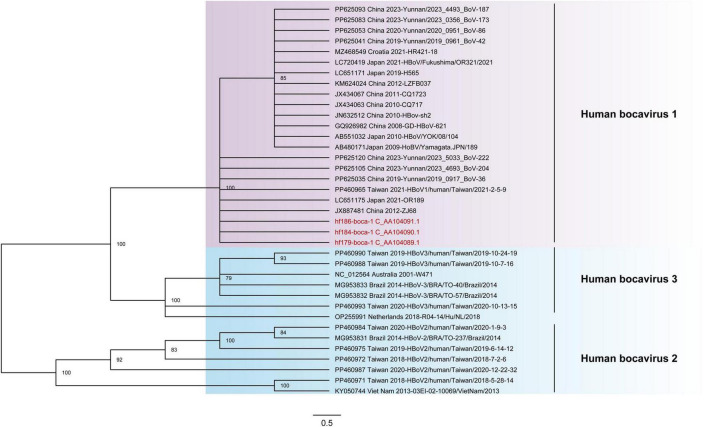
The phylogenetic analysis of HBoVs identified in this study. The phylogenetic trees were constructed based on the nucleotide sequences of NS1 coding region that included the reference strains of three different genotypes. Viruses identified in this study were marked in red font.

In this study, three contigs of HBoVs were obtained from libraries (Humanfe179,184 and 186). These were named hf179-boca-1, hf184-boca-1, and hf186-boca-1, respectively (Supplementary Table 2). The genomes of hf179-boca-1, hf184-boca-1, and hf186-boca-1 are 857nt, 5346 nt, and 5361 nt in length, respectively. The hf179-boca-1 contains a single open reading frame (ORF1), while both the hf184-boca-1 and hf186-boca-1 contain three open reading frames (ORF1, ORF2 and ORF3). The lengths of ORF1 are 857, 1920, and 1920 nt encoding 285, 639, and 639 aa of NS1, respectively, which participate in viral replication and regulate the metabolism of host cells. The lengths of ORF2 and ORF3 in hf184-boca-1 and hf186-boca-1 are 660 nt and 2016 nt encoding 220 aa NP1 and 671 aa of VP1, distinctly. BLASTn search in NCBI revealed that both hf179-boca-1 and hf184-boca-1 possessed the highest degree of nucleotide identity (100.00%) with the strain Yunnan/2019_0917_BoV-36 (PP625035) isolated from China in 2019. The hf186-boca-1 shared the highest degree of nucleotide identity (100.00%) with the strain HBoV/Fukushima/OR321/2021 (LC720419), which was isolated from Japan in 2021. Phylogenetic trees were constructed based on the nucleotide sequence of the NS1 gene, incorporating the reference strains of three HBoVs genotypes. The results indicated that these three bocaviruses clustered with HBoV 1 strains, forming a branch (Figure 3).

### 3.4 Human astrovirus belonging to *Astroviridae*

**FIGURE 4:**
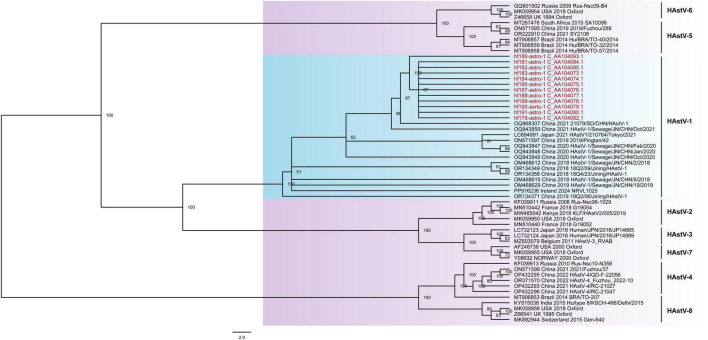
The phylogenetic analysis of HAstVs identified in this study. The phylogenetic trees were constructed based on the nucleotide sequences of ORF2 coding region that included the reference strains of eight different genotypes. Viruses identified in this study were marked in red font.

In this study, 12 nearly complete genomes of human astroviruses (HAstVs) were obtained from libraries (Humanfe179 to 185, 187 to 191) (Supplementary Table 2). All of those HAstVs have three open reading frames (ORF1a, ORF1b and ORF2). The genome lengths vary from 6100 to 6827 nt, and the GC content ranges from 44.3 to 47.8%. The lengths of ORF1a range from 2808 to 2809 nt, encoding 935 aa to 936 aa of the putative serine protease. The lengths of ORF1b are between 1548 and 1560 nt, which encode 515 aa to 519 aa of the RNA-dependent RNA polymerase. Regarding ORF2, its lengths range from 2140 to 2364 nt, encoding 713 to 787 aa of the capsid protein. Sequence analysis was performed using BLASTn search, and the phylogenetic trees were constructed based on the nucleotide sequence of the ORF2, incorporating the reference strains of eight distinct genotypes of HAstVs. This analysis demonstrated that these 12 HAstV genomes exhibited the highest nucleotide identity of 99.5% to the strain 21079/SD/CHN/HAstV-1 (OQ968307), which was isolated from Jinan, China in 2021 and clustered with human astrovirus 1 strains to form a branch (Figure 4).

### 3.5 Norovirus belonging to *Caliciviridae*

**FIGURE 5:**
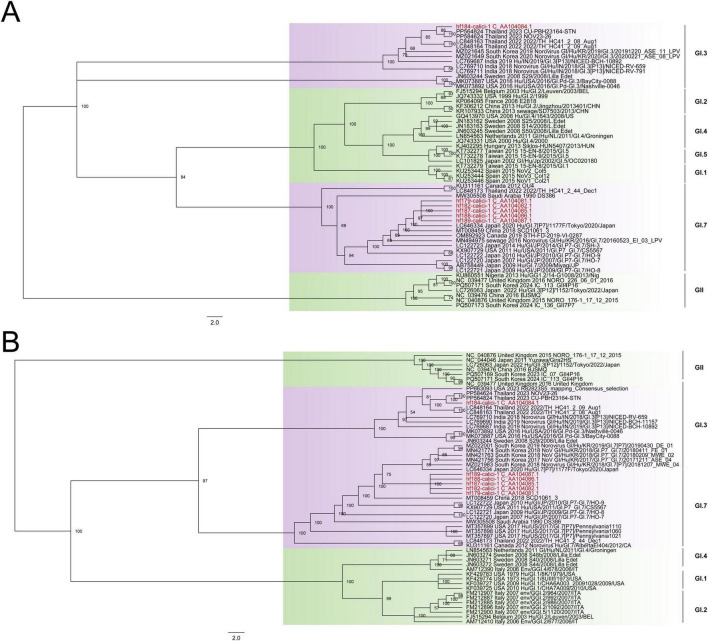
The phylogenetic analysis of NoVs identified in this study based on VP1 and RdRp. **(A)** The phylogenetic trees were constructed based on the nucleotide sequences of VP1 that included the reference strains of seven NoVs genotype. **(B)** The phylogenetic trees were constructed based on the nucleotide sequences of RdRp that included the reference strains of six NoVs genotype. Viruses identified in this study were marked in red font.

Our study identified noroviruses (NoVs) from seven libraries (Humanfe172, 179, 182, 184 and 187–189). A total of six long contigs were obtained, which were named as hf179-calici-1, hf182-calici-1, hf184-calici-1, hf187-calici-1, hf188-calici-1, and hf189-calici-1, respectively (Supplementary Table 2). The genomes of these NoVs range in size from 1810 to 7717 nt. All of these NoVs contain the open reading frames of ORF1 and ORF2, with hf184-calici-1, hf188-calici-1, and hf189-calici-1 additionally containing ORF3. The lengths of ORF1 span from 977 to 5399 nt, encoding 935 to 1798 aa of non-structural polyprotein. The ORF2 of the NoVs in this study are 672–1635 nt in length, encoding 223 to 544 aa of major capsid protein. ORF3 ranges from 426 to 648 nt, encoding the minor structural proteins of 142 to 216 aa. Phylogenetic trees were constructed based on the nucleotide sequences of VP1 (Figure 5A) and RdRp (Figure 5B), with reference strains of seven different genotypes. The result indicated that the six NoVs belonged to two genotypes. The hf184-calici-1 shared nucleotide identity of more than 98.95% to the strain CU-PBH23164-STN (PP564824) isolated from Thailand in 2023, which clustered with norovirus GI.3 strains. The remaining noroviruses exhibited over 99.23% nucleotide identity to the strain Hu/GI.7[P7]/1177F/Tokyo/2020/Japan (LC646334), which was isolated from Japan in 2020, and formed a branch with norovirus GI.7 strains.

### 3.6 Human rhinovirus belonging to *Picornaviridae*

**FIGURE 6:**
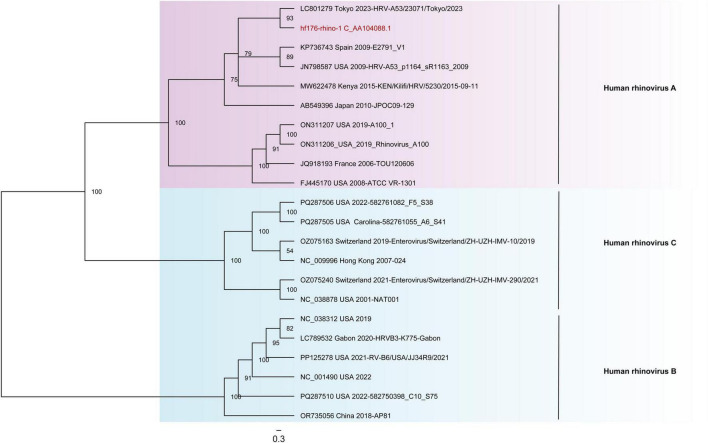
The phylogenetic analysis of HRV identified in this study. The phylogenetic trees were constructed based on the nucleotide sequences of VP4/VP2 coding region that included the reference strains of three different genotypes. Viruses identified in this study were marked in red font.

Here, one contig of HRV was obtained from the library Humanfe176 and was designated as hf176-rhino-1, as documented in Supplementary Table 2. The genome of hf176-rhino-1 is 3481 nt in length. This HRV has one partial open reading frame (ORF1), encoding 949 aa polyprotein. BLASTn search in NCBI indicated that hf176-rhino-1 had the highest nucleotide identity of 98.62% with the strain HRV-A53/23071/Tokyo/2023 (LC801279) isolated from Japan in 2023. The phylogenetic trees were based on the nucleotide sequence of VP4/VP2, which incorporating the reference strains of three HRVs genotypes. The result showed that hf176-rhino-1 clustered with HRV A strains to form a branch (Figure 6).

## 4 Discussion

We collected fecal samples from 121 children afflicted with ARIs and employed the viral metagenomic approach to gain a better understanding of the composition of the gut virome in children with acute respiratory infections. The outcomes demonstrated that the 22 libraries generated a total of 67,118,096 paired-end read sequences with a length of 250 bp. Among these read sequences, 68.52% showed the highest degree of similarity to viruses. Among 47 families, *Podoviridae* had the greatest proportion, reaching 31.1%, followed by *Virgaviridae* (21.5%), *Microviridae* (15.8%), *Astroviridae* (11.3%), and *Siphoviridae* (10.7%). However, a study on the gut viral communities of children with atopic dermatitis and healthy children in the Affiliated Hospital of Jiangsu University indicated that in the gut of healthy children, *Myoviridae* accounted for the highest proportion, followed by *Virgaviridae*, *Caliciviridae*, *Drexlerviridae*, and *Astroviridae* (Lu et al., 2022). There were substantial differences between the two. The same result was found in the comparison with gut virome of healthy Saudi children (El Mouzan et al., 2023). This phenomenon may be associated with a variety of factors, including immune disorders, environmental exposures, and host-pathogen interactions. Environmental factors such as stress influence the composition of the gut virome (Talarico et al., 2024). What’s more, diet is one of the important factors affecting the structure of the gut virome (Zuo et al., 2020). The metabolites originating from the gut microbiome could enhance the type 1 interferon response, providing protection against RSV infection (Antunes et al., 2019) and, somewhat, it affects the composition of viruses in the gut. Conversely, viral infection of the intestine can also influence the body’s immune function and gut microbiota colonization. The gut is an important component of the immune system, with approximately 70% of immune cells residing in gut-associated lymphoid tissue. The invasion of gut viruses can affect intestinal immunity. In SARS-CoV-2 infection patients, spike glycoprotein can induce intestinal barrier dysfunction by binding to angiotensin-converting enzyme II (ACE2) of intestinal epithelial cells (Chen et al., 2020). Persistent or recurrent viral infections can lead to chronic inflammation, increase the risk of autoimmune disease, and bring alterations in the gut virome (Wu et al., 2023). Currently, the interaction between gut virome and the human body involves the invasion of viruses, host immune defense, and synergistic effects of gut microbiota, and the impact of their interaction requires further study.

As is well known, patients with ARIs frequently exhibit gastrointestinal symptoms such as nausea, vomiting, abdominal pain, and diarrhea. These symptoms can also serve as indicators of severe influenza in pediatric patients (Minodier et al., 2015; Shi et al., 2021). Current evidence suggests that gastrointestinal symptoms result from systemic immune activation, and the disruption of the balance of gut microbiota. Notably, respiratory influenza infections induce not only pulmonary immune damage but also intestinal immune dysregulation via the gut-lung axis, as evidenced by impaired epithelial barrier integrity and exacerbated mucosal inflammatory response (Wang et al., 2014). Concurrently, respiratory viral infections promote gut microbiota remodeling, characterized by a decrease in the abundance of immunomodulatory commensals such as *segmented filamentous bacteria* (SFB) and *Lactobacillus/Lactococcus*, alongside expansion of opportunistic pathogens like *Enterobacteriaceae* (Wang et al., 2014). Moreover, influenza A virus (IAV) infection is associated with increased proportions of *Proteobacteria* and *Firmicutes* and decreased *Bacteroidetes* in the gut (Yildiz et al., 2018). The increase in *Proteobacteria* is mediated by type I interferon (IFN), which not only promotes the consumption of obligate anaerobes but also increases the susceptibility to secondary *Salmonella* colitis (Deriu et al., 2016). Importantly, emerging evidence underscores the role of concurrent intestinal infections as an additional contributor to gastrointestinal symptoms in ARIs. In a prospective study on clinical and virological factors associated with gastrointestinal symptoms in patients with acute respiratory infections, it was pointed out that intestinal pathogens were present in the fecal samples of acute respiratory tract infections patients, which could be used to explain the presence of gastrointestinal symptoms in ARI patients (Minodier et al., 2017). This was confirmed by the finding of human adenovirus, human bocavirus, human astrovirus, norovirus, and HRV in feces in the present study. Among them, human astrovirus and norovirus were detected at relatively high levels in the study, and human adenovirus, human bocavirus, and HRV were detected in individual libraries.

It should be noted that human adenovirus, human bocavirus, and HRV are present not only in the intestinal tract, causing intestinal infections, but also in the respiratory tract. In the research on respiratory virome, HAdV (Cui et al., 2024), HBoV (Li Z. et al., 2021), and HRV (Ogunbayo et al., 2022) were detected in the respiratory tract of children and were associated with ARIs. Human bocavirus has been found to be prevalent in both fecal or nasal swab samples of patients with gastrointestinal and respiratory symptoms. In half of the cases of HBoV1-positive respiratory infections, HBoV1 was identified in both feces and nasal swabs; however, it was also observed that when HBoV1 was present in feces, usually gastroenteritis viruses were typically detected as well (Paloniemi et al., 2014). Therefore, it is hypothesized that bocaviruses in the intestinal tract might have originated from the respiratory tract, but the relationship between this and the gastrointestinal symptoms of patients with ARIs remains unclear. The rhinoviruses identified in this study were HRV-A. In a study on the genetic diversity and epidemiology of children with severe ARIs in Guangzhou City, the majority of detected HRVs were found to be HRV-A and HRV-C (Li W. et al., 2021). In a study of 734 fecal samples from children with gastroenteritis, HRV - A was detected in only two samples (Lau et al., 2012). Unfortunately, our study did not record respiratory secretions simultaneously and did not count whether there were accompanying gastrointestinal symptoms. Thus, it is impossible to determine whether the human adenovirus, human bocavirus, and HRV identified in this study entered the gastrointestinal tract by swallowing respiratory secretions and induced gastrointestinal symptoms.

We also elucidated the genetic characteristics and possible epidemiological scenarios of the five eukaryotic viruses mentioned above. The human astrovirus detected in this study belongs to HAstV-1, sharing more than 99% nucleotide identity with the strains identified in Jinan, Xinjiang, and Fuzhou of China. Moreover, HAstV-1 has been frequently detected in children with acute gastroenteritis in Shandong (Huang et al., 2021), Guangzhou (Luo et al., 2021), Shanghai (Wu et al., 2020), and Beijing (Guo et al., 2010). Therefore, we inferred that HAstV-1 was a common genotype of astrovirus infection in the Chinese population. In China, GII.3, and GII.4 were the major genotypes of norovirus infection (Zhou et al., 2020). We identified a total of six norovirus strains, five of which belonged to the GI.7 genotype and one to the GI.3 genotype. In a survey of 4,123 fecal samples from children with non-cholera diarrhea in Shanghai, Hangzhou, Chongqing, and Tianjin, China, during 2008 - 2009, only three samples were of the GI.3 genotype (Zeng et al., 2012). In the genotyping and traceability analyses of noroviruses in Yantai City, Shandong Province, China, from 2017 to 2019, only one sample was identified as the GI.7 genotype (Sun et al., 2023). The norovirus GI.3 and GI.7 genotypes identified in this study were not common genotypes associated with norovirus-associated acute gastroenteritis. Multiple strains of GI.7 were discovered for the first time in the same region of China. The HAdV detected in this study exhibited more than 99% nucleotide identity with human adenovirus C strains from several countries and regions, including the United States, Canada, Germany, Russia, and Argentina. Human adenovirus C was widespread in Brazil (Alves et al., 2024), China (Lu et al., 2023; Wang et al., 2024), and Thailand (Sriwanna et al., 2013), suggesting that the human adenovirus C strains may be globally prevalent. Nevertheless, it was important to note that the prevalence of human adenovirus C was higher in the respiratory tract than the intestinal tract (Sriwanna et al., 2013). The bocavirus we detected belongs to HBoV1, sharing over 99% nucleotide identity with human bocaviruses detected in Yunnan, Jiangsu, Taiwan, Guangdong, and Beijing of China. Previous studies on the prevalence of HBoV infection in patients with acute gastroenteritis in Taiwan (Chiu et al., 2024) and Chengdu (Zhou et al., 2017) found that HBoV1 was relatively common. Furthermore, HBoV1 accounted for a certain proportion of respiratory infections in Chinese children (Wang et al., 2022; Sun et al., 2025). Similar to HBoV1, HRV A was prevalent in patients with both respiratory and gastrointestinal tract infections (Harvala et al., 2012; Chansaenroj et al., 2017). This indicated that HBoV 1 and HRV A were both commonly found in patients with respiratory and gastrointestinal tract infections.

In conclusion, this study provided an overview of the virome present in the gut of children with ARIs and analyzed possible reasons for differences from healthy children in previous studies. In addition, we identified human adenovirus, human bocavirus, human astrovirus, norovirus, and HRV and elucidated their genetic characteristics and potential epidemic scenarios. Further investigation is necessary to determine whether HAdV, HBoV, and HRV detected in the gut originate from the swallowed respiratory secretions. This study has enhanced our understanding of gut viruses of children with ARIs and provides a new perspective for the analysis of the interaction between the respiratory tract and the gastrointestinal tract.

## Data Availability

The original sequence data analyzed in this study have been deposited in the Genome Sequence Archive (Genomics, Proteomics & Bioinformatics 2021) at the National Genomics Data Centre (Nucleic Acids Res 2022), China National Centre for Bioinformation, Beijing Institute of Genomics, and Chinese Academy of Sciences (GSA: CRA021512). It is also publicly available at https://ngdc.cncb.ac.cn/gsa. The genome reported in the current study has been stored in GenBase at the National Genomics Data Centre, Beijing Institute of Genomics, Chinese Academy of Sciences, and China National Centre for Bioinformation under accession numbers C_AA104072.1–C_AA104095.1, which are accessible to the public: https://ngdc.cncb.ac.cn/genbase. The accession numbers are listed in Supplementary Table 2.

## References

[B1] Altschul S. Madden T. Schäffer A. Zhang J. Zhang Z. Miller W. (1997). Gapped BLAST and PSI-BLAST: A new generation of protein database search programs. *Nucleic Acids Res.* 25 3389–3402. 10.1093/nar/25.17.3389 9254694 PMC146917

[B2] Alves J. Teixeira D. Siqueira J. Deus D. Oliveira D. Ferreira J. (2024). Epidemiology and molecular detection of human adenovirus and non-polio enterovirus in fecal samples of children with acute gastroenteritis: A five-year surveillance in northern Brazil. *PLoS One* 19:e0296568. 10.1371/journal.pone.0296568 39093896 PMC11296658

[B3] Antunes K. Fachi J. de Paula R. da Silva E. Pral L. Dos Santos A. Á (2019). Microbiota-derived acetate protects against respiratory syncytial virus infection through a GPR43-type 1 interferon response. *Nat. Commun.* 10:3273. 10.1038/s41467-019-11152-6 31332169 PMC6646332

[B4] Bender R. Sirota S. Swetschinski A. (2024). Global, regional, and national incidence and mortality burden of non-COVID-19 lower respiratory infections and aetiologies, 1990-2021: A systematic analysis from the Global burden of disease study 2021. *Lancet Infect. Dis.* 24 974–1002. 38636536 10.1016/S1473-3099(24)00176-2 PMC11339187

[B5] Capobianchi M. Giombini E. Rozera G. (2013). Next-generation sequencing technology in clinical virology. *Clin. Microbiol. Infect.* 19 15–22. 10.1111/1469-0691.12056 23279287

[B6] Chansaenroj J. Tuanthap S. Thanusuwannasak T. Duang-In A. Klinfueng S. Thaneskongtong N. (2017). Human enteroviruses associated with and without diarrhea in Thailand between 2010 and 2016. *PLoS One* 12:e0182078. 10.1371/journal.pone.0182078 28750058 PMC5531555

[B7] Chen H. Zou T. Xuan B. Yan Y. Yan T. Shen C. (2020). Single cell transcriptome revealed SARS-CoV-2 entry genes enriched in colon tissues and associated with coronavirus infection and cytokine production. *Signal. Transduct. Target Ther.* 5:121. 10.1038/s41392-020-00237-0 32641705 PMC7340775

[B8] Chiu S. Yu Y. Hsieh L. Chen Y. Lu Y. Chang J. (2024). Human bocavirus circulating in patients with acute gastroenteritis in Taiwan, 2018-2022. *Viruses* 16:1630. 10.3390/v16101630 39459962 PMC11512290

[B9] Cui S. Guo R. Chen C. Zhang Y. Meng J. Liu L. (2024). Next-generation sequencing for characterizing respiratory tract virome and improving detection of viral pathogens in children with Pneumonia. *Influenza Other Respir. Viruses.* 18:e13362. 10.1111/irv.13362 39118486 PMC11310556

[B10] Deng X. Naccache S. Ng T. Federman S. Li L. Chiu C. (2015). An ensemble strategy that significantly improves de novo assembly of microbial genomes from metagenomic next-generation sequencing data. *Nucleic Acids Res.* 43:e46. 10.1093/nar/gkv002 25586223 PMC4402509

[B11] Deriu E. Boxx G. He X. Pan C. Benavidez S. Cen L. (2016). Influenza virus affects intestinal microbiota and secondary *Salmonella* Infection in the gut through type i interferons. *PLoS Pathog.* 12:e1005572. 10.1371/journal.ppat.1005572 27149619 PMC4858270

[B12] Edgar R. C. (2004). MUSCLE: Multiple sequence alignment with high accuracy and high throughput. *Nucleic Acids Res.* 32 1792–1797. 10.1093/nar/gkh340 15034147 PMC390337

[B13] El Mouzan M. Assiri A. Al Sarkhy A. Alasmi M. (2023). Gut virome profile in healthy Saudi children. *Saudi J. Gastroenterol.* 29 171–176. 10.4103/sjg.sjg_444_22 37313947 PMC10358797

[B14] Gauthier N. Chorlton S. Krajden M. Manges A. (2023). Agnostic sequencing for detection of viral pathogens. *Clin. Microbiol. Rev.* 36:e0011922. 10.1128/cmr.00119-22 36847515 PMC10035330

[B15] Guo L. Xu X. Song J. Wang W. Wang J. Hung T. (2010). Molecular characterization of astrovirus infection in children with diarrhea in Beijing, 2005-2007. *J. Med. Virol.* 82 415–423. 10.1002/jmv.21729 20087940 PMC7166319

[B16] Harvala H. McIntyre C. McLeish N. Kondracka J. Palmer J. Molyneaux P. (2012). High detection frequency and viral loads of human rhinovirus species A to C in fecal samples; diagnostic and clinical implications. *J. Med. Virol.* 84 536–542. 10.1002/jmv.23203 22246843

[B17] Huang D. Wang Z. Zhang F. Wang T. Zhang G. Sai L. (2021). Molecular and clinical epidemiological features of human astrovirus infections in children with acute gastroenteritis in Shandong province, China. *J. Med. Virol.* 93 4883–4890. 10.1002/jmv.26995 33811682

[B18] Huson D. Beier S. Flade I. Górska A. El-Hadidi M. Mitra S. (2016). MEGAN community edition - interactive exploration and analysis of large-scale microbiome sequencing data. *PLoS Comput. Biol.* 12:e1004957. 10.1371/journal.pcbi.1004957 27327495 PMC4915700

[B19] Jain S. Williams D. Arnold S. Ampofo K. Bramley A. Reed C. (2015). Community-acquired pneumonia requiring hospitalization among U.S. children. *N. Engl. J. Med.* 372 835–845. 10.1056/NEJMoa1405870 25714161 PMC4697461

[B20] Kumar S. Stecher G. Li M. Knyaz C. Tamura K. (2018). MEGA X: Molecular evolutionary genetics analysis across computing platforms. *Mol. Biol. Evol.* 35 1547–1549. 10.1093/molbev/msy096 29722887 PMC5967553

[B21] Langmead B. Salzberg S. (2012). Fast gapped-read alignment with Bowtie 2. *Nat. Methods* 9 357–359. 10.1038/nmeth.1923 22388286 PMC3322381

[B22] Lau S. Yip C. Lung D. Lee P. Que T. Lau Y. (2012). Detection of human rhinovirus C in fecal samples of children with gastroenteritis. *J. Clin. Virol.* 53 290–296. 10.1016/j.jcv.2012.01.008 22317907 PMC7108355

[B23] Leal Rodríguez C. Shah S. Rasmussen M. Thorsen J. Boulund U. Pedersen C. (2024). The infant gut virome is associated with preschool asthma risk independently of bacteria. *Nat. Med.* 30 138–148. 10.1038/s41591-023-02685-x 38102298

[B24] Li W. Yu B. Zhou J. Wang Y. Xue B. Pan J. (2021). Genetic diversity and epidemiology of human rhinovirus among children with severe acute respiratory tract infection in Guangzhou, China. *Virol. J.* 18:174. 10.1186/s12985-021-01645-6 34425845 PMC8382100

[B25] Li Z. Zhang H. Ren L. Lu Q. Ren X. Zhang C. (2021). Etiological and epidemiological features of acute respiratory infections in China. *Nat. Commun.* 12:5026. 10.1038/s41467-021-25120-6 34408158 PMC8373954

[B26] Lu L. Jia R. Zhong H. Duan S. Xu M. Su L. (2023). Surveillance and epidemiological characterization of human adenovirus infections among outpatient children with acute gastroenteritis during the COVID-19 epidemic in Shanghai, China. *Virol. J.* 20:133. 10.1186/s12985-023-02105-z 37344873 PMC10286426

[B27] Lu X. Wang H. Zhang J. Jin K. Ma L. Wang Y. (2022). Comparison of gut viral communities in atopic dermatitis and healthy children. *Front. Med (Lausanne).* 9:835467. 10.3389/fmed.2022.835467 35265642 PMC8899399

[B28] Lu Z. Zhou H. Wu W. Fu T. Yan M. He Z. (2021). Alterations in the composition of intestinal DNA virome in patients with COVID-19. *Front. Cell. Infect. Microbiol.* 11:790422. 10.3389/fcimb.2021.790422 34900762 PMC8653907

[B29] Luo X. Deng J. Mu X. Yu N. Che X. (2021). Detection and characterization of human astrovirus and sapovirus in outpatients with acute gastroenteritis in Guangzhou, China. *BMC Gastroenterol.* 21:455. 10.1186/s12876-021-02044-5 34861832 PMC8642882

[B30] Minodier L. Charrel R. N. Ceccaldi P. E. Van Der Werf S. Blanchon T. Hanslik T. (2015). Prevalence of gastrointestinal symptoms in patients with influenza, clinical significance, and pathophysiology of human influenza viruses in faecal samples: What do we know? *Virol. J.* 12:215. 26651485 10.1186/s12985-015-0448-4 PMC4676820

[B31] Minodier L. Masse S. Capai L. Blanchon T. Ceccaldi P. van der Werf S. (2017). Clinical and virological factors associated with gastrointestinal symptoms in patients with acute respiratory infection: A two-year prospective study in general practice medicine. *BMC Infect. Dis.* 17:729. 10.1186/s12879-017-2823-9 29166867 PMC5700681

[B32] Ogunbayo A. Mogotsi M. Sondlane H. Nkwadipo K. Sabiu S. Nyaga M. (2022). Metagenomic analysis of respiratory RNA virome of children with and without severe acute respiratory infection from the free state, south africa during COVID-19 pandemic reveals higher diversity and abundance in summer compared with winter period. *Viruses* 14:2516. 10.3390/v14112516 36423125 PMC9692838

[B33] Ogunbayo A. Mogotsi M. Sondlane H. Sabiu S. Nyaga M. (2023). Metagenomics characterization of respiratory viral RNA pathogens in children under five years with severe acute respiratory infection in the Free State, South Africa. *J. Med. Virol.* 95:e28753. 10.1002/jmv.28753 37212321 PMC10952945

[B34] Paloniemi M. Lappalainen S. Salminen M. Kätkä M. Kantola K. Hedman L. (2014). Human bocaviruses are commonly found in stools of hospitalized children without causal association to acute gastroenteritis. *Eur. J. Pediatr.* 173 1051–1057. 10.1007/s00431-014-2290-x 24590657

[B35] Ronquist F. Teslenko M. van der Mark P. Ayres D. Darling A. Höhna S. (2012). MrBayes 3.2: Efficient Bayesian phylogenetic inference and model choice across a large model space. *Syst. Biol.* 61 539–542. 10.1093/sysbio/sys029 22357727 PMC3329765

[B36] Shi Y. Chen W. Zeng M. Shen G. Sun C. Liu G. (2021). Clinical features and risk factors for severe influenza in children: A study from multiple hospitals in Shanghai. *Pediatr. Neonatol.* 62 428–436. 10.1016/j.pedneo.2021.05.002 34103261

[B37] Sriwanna P. Chieochansin T. Vuthitanachot C. Vuthitanachot V. Theamboonlers A. Poovorawan Y. (2013). Molecular characterization of human adenovirus infection in Thailand, 2009-2012. *Virol. J.* 10:193. 10.1186/1743-422X-10-193 23758792 PMC3693972

[B38] Sun Y. Jiang L. Chen Y. Liu Z. Zhang M. Zhao X. (2025). Prevalence and molecular characterization of human bocavirus-1 in children and adults with influenza-like illness from Kunming, Southwest China. *Microbiol. Spectr.* 13:e0156424. 10.1128/spectrum.01564-24 39660928 PMC11705858

[B39] Sun Z. Niu P. Jin M. Zhang R. Gao Q. Wang H. (2023). Genotyping and traceability analysis of norovirus in Yantai between 2017 and 2019. *J. Med. Virol.* 95:e29220. 10.1002/jmv.29220 37947460

[B40] Talarico F. Tilocca B. Spagnuolo R. Abenavoli L. Luzza F. Roncada P. (2024). The effects of stress on gut virome: Implications on infectious disease and systemic disorders. *Microbiologyopen* 13:e1434. 10.1002/mbo3.1434 39311537 PMC11418023

[B41] Wang F. De R. Han Z. Xu Y. Zhu R. Sun Y. (2024). High-frequency recombination of human adenovirus in children with acute respiratory tract infections in Beijing, China. *Viruses* 16:828. 10.3390/v16060828 38932121 PMC11209268

[B42] Wang J. Li F. Wei H. Lian Z. Sun R. Tian Z. (2014). Respiratory influenza virus infection induces intestinal immune injury via microbiota-mediated Th17 cell-dependent inflammation. *J. Exp. Med.* 211 2397–2410. 10.1084/jem.20140625 25366965 PMC4235643

[B43] Wang W. Guan R. Liu Z. Zhang F. Sun R. Liu S. (2022). Epidemiologic and clinical characteristics of human bocavirus infection in children hospitalized for acute respiratory tract infection in Qingdao, China. *Front. Microbiol.* 13:935688. 10.3389/fmicb.2022.935688 36033842 PMC9399728

[B44] Wu L. Teng Z. Lin Q. Liu J. Wu H. Kuang X. (2020). Epidemiology and genetic characterization of classical human astrovirus infection in Shanghai, 2015-2016. *Front. Microbiol.* 11:570541. 10.3389/fmicb.2020.570541 33101242 PMC7546348

[B45] Wu R. Mumtaz M. Maxwell A. Isaacs S. Laiho J. Rawlinson W. (2023). Respiratory infections and type 1 diabetes: Potential roles in pathogenesis. *Rev. Med. Virol.* 33:e2429. 10.1002/rmv.2429 36790804 PMC10909571

[B46] Xiao Y. Wang H. Feng L. Pan J. Chen Z. Wang H. (2020). Fecal, oral, blood and skin virome of laboratory rabbits. *Arch. Virol.* 165 2847–2856. 10.1007/s00705-020-04808-y 33034764 PMC7546134

[B47] Yildiz S. Mazel-Sanchez B. Kandasamy M. Manicassamy B. Schmolke M. (2018). Influenza A virus infection impacts systemic microbiota dynamics and causes quantitative enteric dysbiosis. *Microbiome* 6:9. 10.1186/s40168-017-0386-z 29321057 PMC5763955

[B48] Zeng M. Xu X. Zhu C. Chen J. Zhu Q. Lin S. (2012). Clinical and molecular epidemiology of norovirus infection in childhood diarrhea in China. *J. Med. Virol.* 84 145–151. 10.1002/jmv.22248 22028199

[B49] Zhang W. Li L. Deng X. Kapusinszky B. Delwart E. (2014a). What is for dinner? Viral metagenomics of US store bought beef, pork, and chicken. *Virology* 468-470 303–310. 10.1016/j.virol.2014.08.025 25217712 PMC4252299

[B50] Zhang W. Li L. Deng X. Kapusinszky B. Pesavento P. Delwart E. (2014b). Faecal virome of cats in an animal shelter. *J. Gen. Virol.* 95 2553–2564. 10.1099/vir.0.069674-0 25078300 PMC4202271

[B51] Zhou H. Wang S. von Seidlein L. Wang X. (2020). The epidemiology of norovirus gastroenteritis in China: Disease burden and distribution of genotypes. *Front. Med.* 14:1–7. 10.1007/s11684-019-0733-5 31823287 PMC8320309

[B52] Zhou T. Chen Y. Chen J. Hu P. Zheng T. Xu X. (2017). Prevalence and clinical profile of human bocavirus in children with acute gastroenteritis in Chengdu, West China, 2012-2013. *J. Med. Virol.* 89 1743–1748. 10.1002/jmv.24787 28198551

[B53] Zuo T. Sun Y. Wan Y. Yeoh Y. Zhang F. Cheung C. (2020). Human-Gut-DNA virome variations across geography, ethnicity, and urbanization. *Cell Host Microbe* 28 741–751.e4. 10.1016/j.chom.2020.08.005 32910902

